# Erythema nodosum leprosum after allergen immunotherapy as initial presentation of lepromatous leprosy treated with novel multidrug regimen

**DOI:** 10.1016/j.jdcr.2023.09.012

**Published:** 2023-09-27

**Authors:** Catherine B. Xie, Barbara M. Stryjewska, Jennifer McNiff, Bethel Shiferaw

**Affiliations:** a Department of Dermatology, Yale University School of Medicine, New Haven, Connecticut; b Department of Internal Medicine, St. Mary’s Hospital, Waterbury, Connecticut; c National Hansen’s Disease Program, Baton Rouge, Louisiana; d Department of Pathology, Yale University School of Medicine, New Haven, Connecticut; e Department of Infectious Disease, St. Mary’s Hospital, Waterbury, Connecticut

## Introduction

Although <5% of the world’s population is seemingly susceptible to *Mycobacterium leprae* infection*,* host genetics and environmental factors, including immunodeficient or immunosuppressed states, predispose toward the severe disease spectral state of the lepromatous (multibacillary) leprosy.^1^ We present a case of a patient undergoing allergen immunotherapy, who suddenly developed erythema nodosum leprosum (ENL) as the initial manifestation of lepromatous leprosy (LL) and was treated with a novel monthly multidrug antibiotic regimen in combination with immunomodulatory therapy. This is a rare case report of a difficult-to-treat leprosy immune reaction that developed after a preceding immunologic event of allergen immunotherapy.

## Case report

**Fig 1:**
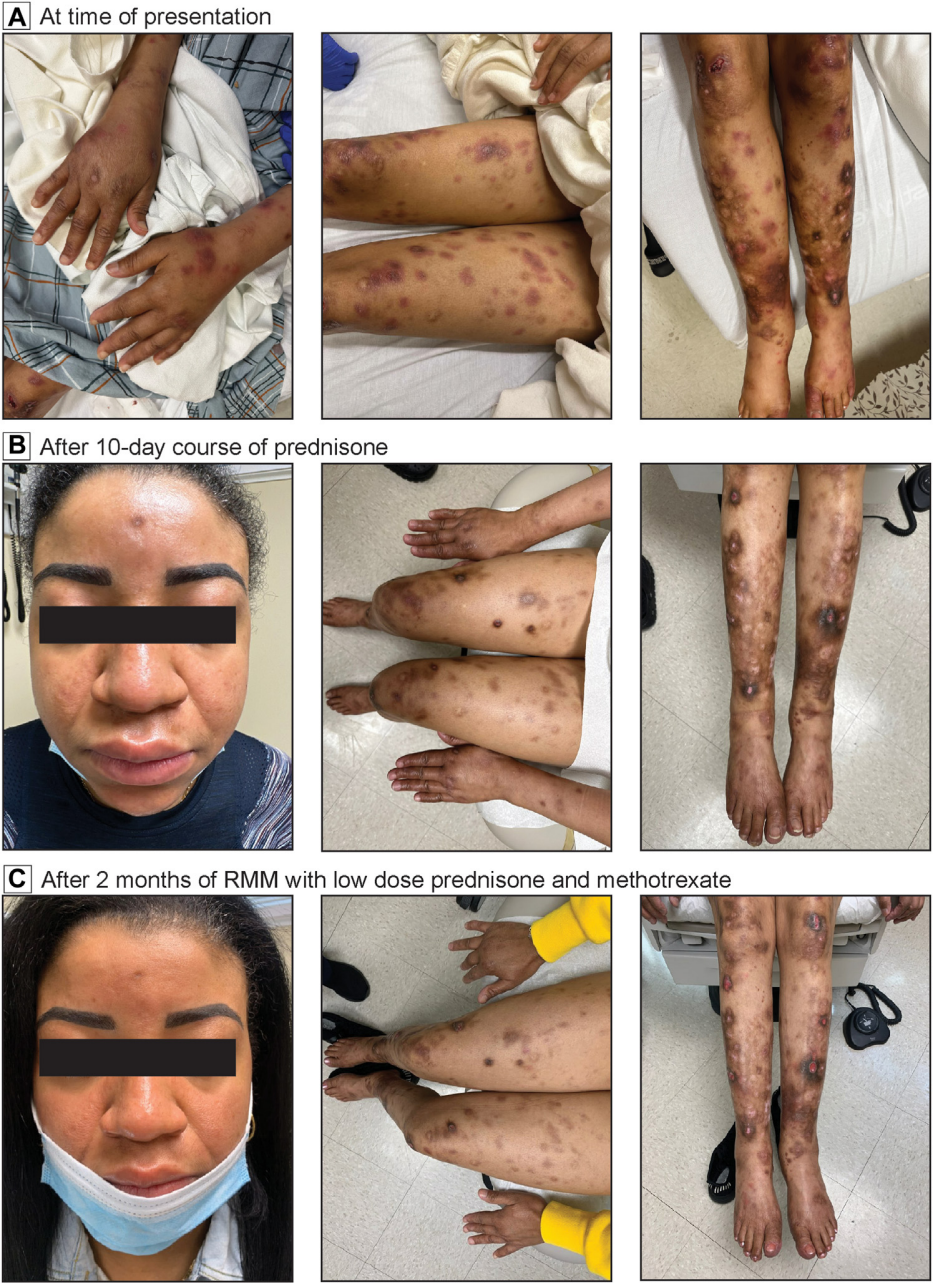
Clinical images showing acute erythema nodosum leprosum lesions on background of lepromatous lesions. **A,** Crops of erythematous inflamed papules and nodules on patient’s upper and lower extremities with brown hyperpigmented macules and papules with central ulcerations associated with edema. **B,** Follow up after 10-day course of prednisone (1 mg/kg). **C,** 2-month follow up on monthly rifampin, moxifloxacin, and minocycline (RMM) therapy and combination low-dose prednisone and methotrexate.

**Fig 2:**
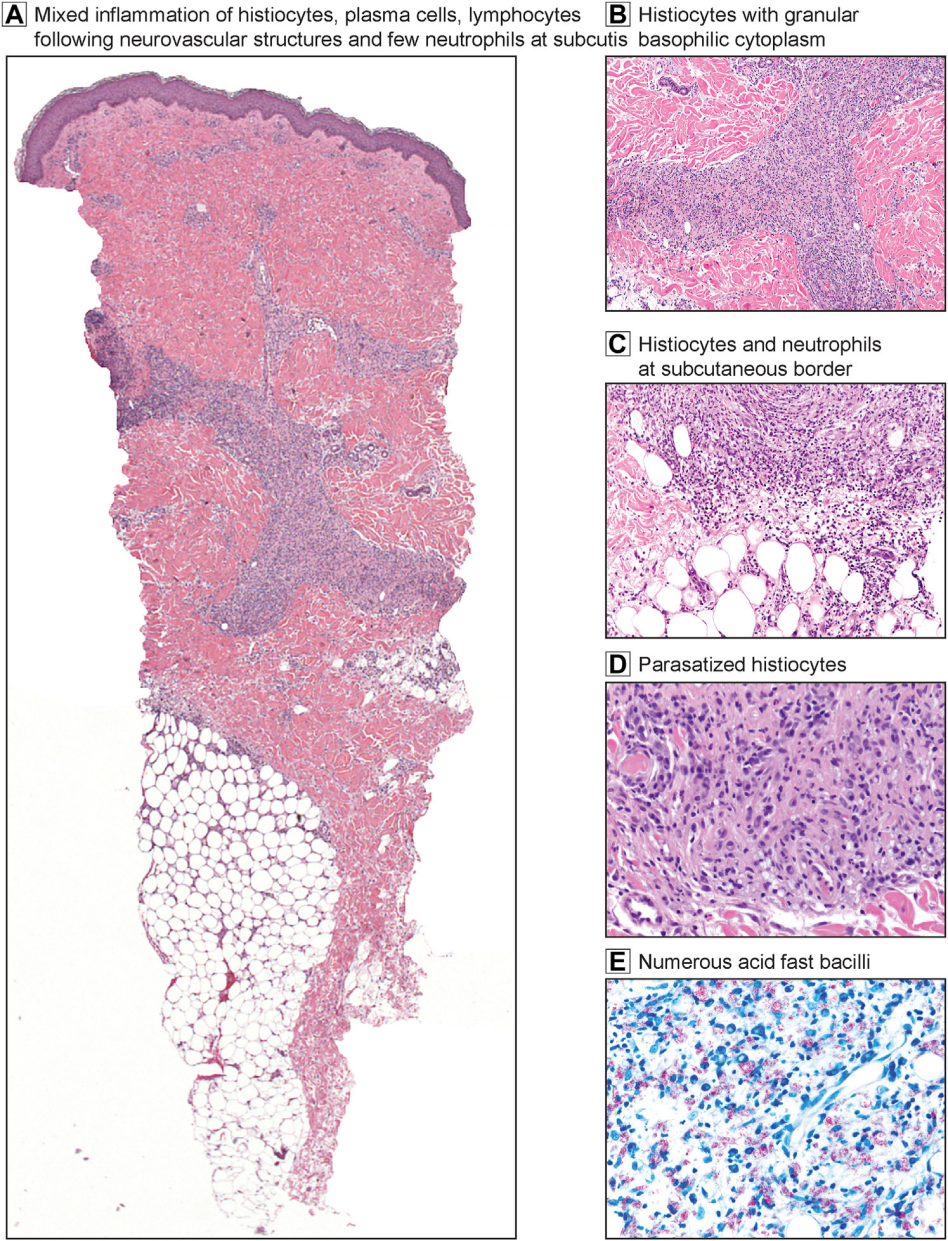
Skin biopsy from right thigh showing mixed inflammatory infiltrate of erythema nodosum leprosum with high-bacillary load. **A,** Low-power view showing inflammation following neurovascular structures, composed of foamy histiocytes, plasma cells, lymphocytes, and a few neutrophils. A few neutrophils are noted at the border of dermis and subcutis. (Hematoxylin-eosin stain; original magnification: 20×.) **B,** High-power view of the mixed inflammation showing histiocytes with granular basophilic cytoplasm. (Hematoxylin-eosin stain; original magnification: 100×.) **C,** There is modest mixed inflammation containing histiocytes with blue cytoplasm and neutrophils at the subcutaneous border spilling into the fat. (Hematoxylin-eosin stain; original magnification: 200×.) **D,** Granular blue cytoplasm of histiocytes parasitized by acid-fast bacilli. (Hematoxylin-eosin stain; Original magnification: 400×.) **E,** Numerous acid-fast bacilli are located in histiocytes on Fite stain. (Fite stain; original magnification: 400×.)

A 36-year-old woman who emigrated from the Dominican Republic several years prior with medical history of atopic dermatitis, allergic rhinitis, and dust mite hypersensitivity presented with a sudden eruption of tender red papules and nodules with central ulcerations distributed on the bilateral upper and lower extremities, including feet and hands, with associated edema, high-grade fever, and malaise after 4 weeks of subcutaneous allergen immunotherapy (Fig 1, *A*). No numbness, loss of sensation, or muscle weakness were found on examination. Skin biopsy demonstrated mixed inflammation of parasitized foamy histiocytes, plasma cells, and lymphocytes following neurovascular structures with a few neutrophils at the subcutis (Fig 2, *A*-*D*). Notably, clusters of acid-fast bacilli within histiocytes resembling “globi” were observed on Fite stain (Fig 2, *E*) and Acid-fast bacillus stain. Acid-fast bacillus culture of the skin biopsy for 8 weeks was negative. Polymerase chain reaction confirmed *M. leprae* detection with no drug-resistant mutation genes detected. These findings along with clinical presentation of the typical skin lesions found symmetrically on extremities with systemic symptoms and pathologic features of scant neutrophilic infiltrate at the subcutaneous border in setting of histiocytes with high lepra bacillary load, were compatible with acute-onset ENL, a type 2 leprosy reaction, as the initial manifestation of lepromatous multibacillary leprosy.

After consultation with the National Hansen’s Disease Program, monthly multidrug therapy of rifampin, moxifloxacin, and minocycline (RMM) was chosen to treat the LL over the World Health Organization (WHO) regimen of rifampin, dapsone, and clofazimine. ENL lesions and constitutional symptoms receded expeditiously after 10 days of prednisone (1 mg/kg) (Fig 1, *B*). Along with RMM, combination low-dose prednisone (10 mg daily) and low-dose weekly methotrexate (15 mg weekly) was used to treat the ENL given the patient’s high-reoccurrence probability of immune reaction. Rare new tender ENL papules reoccurred during treatment without cessation of antimicrobial or immunomodulatory therapy (Fig 1, *C*).

## Discussion

We report a case of ENL in a patient with LL that occurred after a preceding immunologic event of allergen immunotherapy. Prior cases of multibacillary leprosy with type 2 reactions after COVID-19 vaccination have been reported.^2^^,^^3^ The mechanistic connection between allergen immunotherapy, LL, and leprosy reactions has never been directly studied but is most likely related to the alteration of the host T-cell response. Patients with LL are unable to control the infection because of a failure to mount an effective antimicrobial cell-mediated immune response against *M. leprae* and exhibit a predominantly Th2 response and humoral immunity.^1^ Allergen immunotherapy targets the Th2 cell pathway by reducing the number of Th2 cells and shifting Th2 cells toward an anergic phenotype in addition to enhancing Th1 responses.^4^, ^5^, ^6^ Enhanced Th1 antimicrobial responses with decreased expression of Th2 cytokines have been observed to predominate and increase during ENL reactions in patients with LL.^7^ Most likely in our case, the preceding immunotherapy induced a switch from a Th2 dominant response to a Th1 cell-dominant immune reaction, triggering the ENL reaction in a patient with previously undetected and undiagnosed leprosy.

Leprosy immune reactions are known to occur before, during, or after therapy and can present a difficult therapeutic challenge. Combination of low-dose prednisone with methotrexate as a steroid-sparing agent has been shown to have equivalent efficacy to high-dose glucocorticoids with better safety profile and lower maintenance dosing.^8^

High rates of adverse effects and poor adherence associated with the WHO multidrug therapy led to the development of a monthly regimen of RMM as an effective alternative treatment for leprosy.^8^, ^9^, ^10^ When compared with the WHO-recommended rifampin, clofazimine, and dapsone, RMM has comparable efficacy with rapid clinical response and decrease in bacilli load in skin.^10^ RMM is operationally less demanding for providers and patients with monthly directly observed therapy. Daily rifampin is a potent inducer of hepatic enzymes that often necessitates increasing the dose of medications, including corticosteroids. Dapsone is associated with rare but serious adverse effects, and clofazimine-induced stigmatizing hyperpigmentation is a leading cause for patient refusal of therapy. Our case uniquely demonstrates type 2 leprosy reaction following allergen immunotherapy in multibacillary leprosy and use of a favorable, effective alternative antimicrobial and immunomodulatory regimen for treatment of severe leprosy and ENL.

## Conflicts of interest

None.
